# Coverage of community case management for malaria through CHWs: a quantitative assessment using primary household surveys of high-burden areas in Chhattisgarh state of India

**DOI:** 10.1186/s12936-020-03285-7

**Published:** 2020-06-22

**Authors:** Samir Garg, Preeti Gurung, Mukesh Dewangan, Prabodh Nanda

**Affiliations:** State Health Resource Centre, Chhattisgarh, Raipur India

**Keywords:** Malaria, Community health workers (CHWs), Community case management, Coverage, Treatment completion, Large-scale, India

## Abstract

**Background:**

Community Case Management of Malaria (CCMM) has been implemented through community health workers (CHWs) in many countries. Existing studies have shown that CHWs can be viable means of implementing CCMM. However, not many studies have examined the coverage under large-scale CCMM programmes. India is a big contributor to global malaria burden. Chhattisgarh is a leading state in India in terms of malaria incidence and mortality. CCMM was implemented on a large scale through the ‘mitanin’ CHWs in rural Chhattisgarh from 2015. Under CCMM, 37,696 CHWs in 84 high-burden administrative blocks of the state were trained and equipped with rapid diagnostic tests (RDT), artemisinin-based combination therapy (ACT) and chloroquine.

**Methods:**

This descriptive quantitative study assesses coverage of CCMM in detection and treatment of Malaria over three rounds of household surveys—2015, 2016 and 2018. Household-interviews covered more than 15,000 individuals in each round, using multi-stage random sampling across the 84 blocks. The main objectives were to find out the coverage in identification and treatment of malaria and the share of CHWs in them. A 15-days recall was used to find out cases of fever and healthcare sought by them.

**Results:**

In 2018, 62% of febrile cases in rural population contacted CHWs. RDT, ACT and chloroquine were available with 96%, 80% and 95% of CHWs, respectively. From 2015 to 2018, the share of CHWs in testing of febrile cases increased from 34 to 70%, while it increased from 28 to 69% in treatment of malaria cases. CHWs performed better than other providers in treatment-completion and administered medication under direct observation to 72% of cases they treated.

**Conclusion:**

This study adds to one of the most crucial but relatively less reported area of CCMM programmes, i.e. the extent of coverage of the total febrile population by CHWs, which subsequently determines the actual coverage of case-management in malaria. Mitanin-CHWs achieved high coverage and treatment-completion rates that were rarely reported in context of large-scale CCMM elsewhere. Close to community, well-trained CHWs with sufficient supplies of rapid tests and anti-malarial drugs can play a key role in achieving the desired coverage in malaria-management.

## Background

The Sustainable Development Goals adopted by United Nations have included the goal to end epidemics of malaria. A target of WHO’s Global Technical Strategy for Malaria (2016–2030) is to reduce malaria-incidence and mortality rates globally by at least 90% by 2030 from the 2015 level. In 2015, there were estimated 212 million malaria cases globally and 90% of them were in Africa, followed by South Asia at 7% [1].

India contributed to around 89% of total malaria cases in the South-East Asian region in 2015 [1]. Within India, Chhattisgarh state reported the second-highest number of malaria cases amongst all states [2]. Chhattisgarh represents around 2% of India’s population, but contributed to 12% of malaria cases in 2014 [2, 3]. The state has 44% of its area under forests and around 31% of population belongs to indigenous tribes [4]. It was termed as a high-burden state for malaria with an annual parasite incidence (API) of 4.72 per 1000 population recorded in 2014 [2]. The infection was unevenly distributed geographically across the state and certain high-transmission districts in Chhattisgarh recorded API greater than 10 per 1000 population in 2014 [2]. More than 80% of reported malaria cases were due to *Plasmodium falciparum* [2].

Chhattisgarh state has implemented a community health worker (CHW) programme since year 2001 [5]. It has 69,991 CHWs known as ‘*mitanin*’, covering a rural population of around 19 million and urban slum population of 2 million [6]. An evaluation of the mitanin-CHW programme in 2010 had found it effective in reducing child under-nutrition and in improving maternal and child health in Chhattisgarh [5].

In 2014, the Department of Health and Family Welfare, Chhattisgarh, decided to implement Community Case Management of Malaria (CCMM) through mitanin-CHWs. The programme involved training and equipping CHWs for this role by building upon their earlier training on malaria [7]. There was a 6-month long preparatory phase in order to bring the stakeholders on-board and carry-out the necessary capacity-building and mobilization. An assessment of a representative sample of CHWs (1106) for their CCMM skills carried out in field in 2016 showed that 98% could carry out correct testing and 88% had adequate skills in treatment [8]. Later, this was confirmed in a practical examination by an external examination board [9].

The CCMM programme was focused on 84 rural blocks with high burden of malaria and which contributed to around 90% of reported malaria cases in the state [10]. The above 84 blocks had 37,696 CHWs, covering a rural population of around 9 million across 9947 villages. Most mitanin-CHWs are residents of the community they cover and the average population covered per CHW was 235 in the above 84 blocks in 2014. The farthest household looked after by a CHW was usually within a kilometre of her residence. The CCMM programme was preceded by a campaign by CHWs for educating communities on malaria and mobilizing them for inter-sector activities for malaria-prevention [7].

**Fig. 1:**
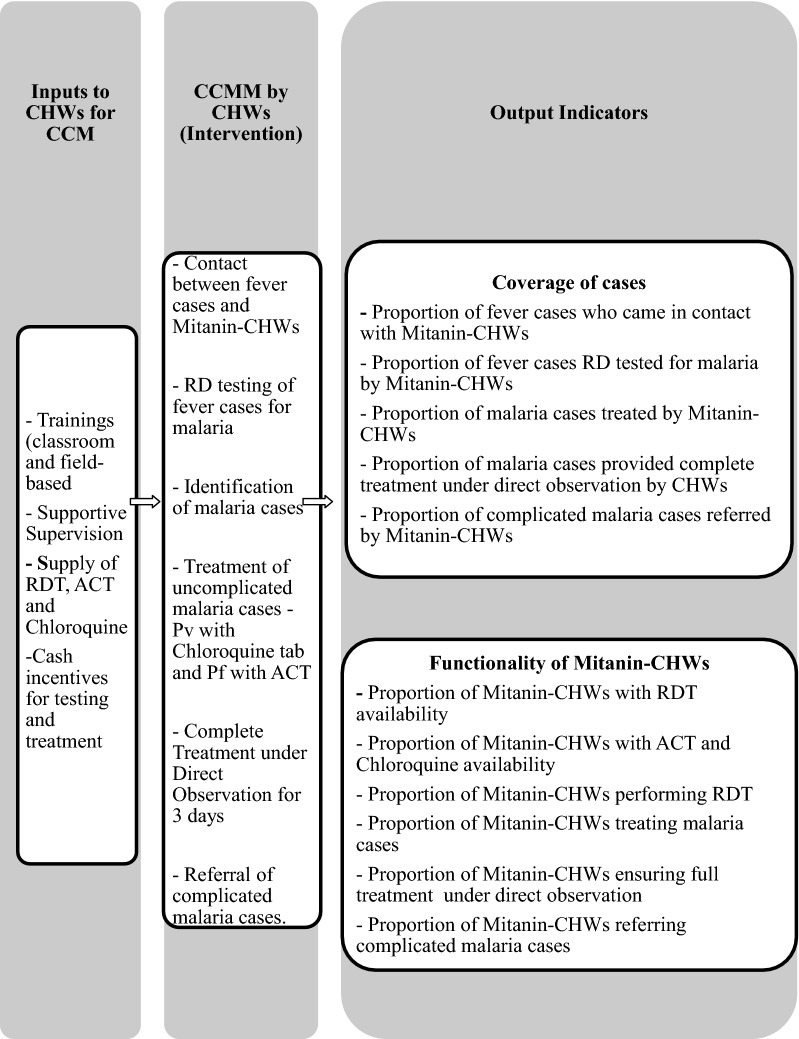
Inputs and output indicators for CCMM intervention in Chhattisgarh

The CCMM intervention involved testing of fever cases in communities by CHWs using bivalent rapid diagnostic tests (RDT) to detect malaria. *Plasmodium vivax* cases thus detected were to be treated with chloroquine tablets and *P. falciparum* cases with artemisinin-based combinations. The intervention was meant to cover children as well as adults. The field-level implementation of the CCMM intervention started from June 2015 onwards after mitanin-CHWs were trained and supplied with RDT and anti-malarial drugs. Incentive of Indian Rupees 23 (around 0.32 US Dollars) per fever case tested was paid to CHWs. The CHWs were trained to ensure treatment under direct observation. For complete treatment, incentive of Indian Rupees 150 (around 2.15 US Dollars) per case treated was paid to CHWs [11]. CHWs were not to take any charges from patients or community. The preparatory phase also included estimating the requirement of RDTs and anti-malarial drugs and making arrangements for their timely procurement. No baseline study was conducted prior to the rolling out of the intervention. Figure 1 exhibits the intervention along with the inputs and the expected output indicators.

There have been many programmes in different low and medium income countries (LMICs) to implement CCMM through CHWs [12]. Existing studies have shown that CHWs can be viable means of implementing CCMM [13–20]. However, most of the existing studies pertain to small-scale interventions [21]. There are not many studies which have examined large-scale programmes covering entire provinces or countries [21]. There has been a study of large-scale CCMM through CHWs in Burkina Faso which shows that the programme was able to achieve a very limited coverage of malaria cases [21]. Another study from Senegal reports on scaled-up CCMM, but the programme was still limited to 861 CHWs [22].

Coverage of population under CCMM, in terms of contact between febrile cases and CHWs has not been studied in India. Treatment completion for malaria in the context of CCMM by CHWs is another area where hardly any studies exist in India. Overall, very limited analysis is available on performance of CHWs inthe context of large-scale programmes on community-management of malaria, including in India. This current study was therefore aimed at assessing performance of a large-scale CCMM programme implemented through mitanin-CHWs in Chhattisgarh state.

## Methods

The term ‘coverage’ was used in this study to denote coverage under the different steps of case management for malaria including—contact, detection, treatment and follow-up for adherence. The indicators of ‘coverage’ as used in this study were:

- Proportion of fever cases who came in contact with mitanin-CHWs
- Proportion of fever cases RD tested for malaria by mitanin-CHWs
- Proportion of malaria cases treated by mitanin-CHWs
- Proportion of malaria cases provided complete treatment under direct observation by CHWs (Adherence).

### Setting and design

For this descriptive study, three rounds of cross-sectional household surveys were carried out in 84 rural blocks of Chhattisgarh state where the CCMM was implemented by the Department of Health through mitanin-CHWs. The first round was carried out in August, 2015, second in November, 2016 and third in November, 2018. The study used multiple rounds in order to capture the evolution of the programme in meeting its key objectives.

Multi-stage random sampling was used covering all 84 blocks. Since one of the aims of the study was to study the share of CHWs in testing of fever cases, an adequate number of fever cases were needed in the sample. A sample requirement of 574 fever cases was calculated for 5% detectable difference at 95% confidence with design effect of 1.5. Assuming 4% incidence of fever (in 15-day recall) and 5 persons per household, there was a need to cover around 14,350 individuals or around 2870 households in each round. The 2018 round covered a bigger sample than earlier rounds. The number of household respondents who participated in the three rounds of surveys and the number of individuals covered, i.e. the members of their households they reported about, is given in Table 1.

**Table 1:** Number of household respondents and population covered in survey

Respondents	August, 2015	November, 2016	November, 2018
Households	3277	3049	4257
Population	15,679	15,023	21,405

The study used descriptive quantitative methods. A structured questionnaire was used for interviewing households and it had a 15-days recall. For each sample household, all members of the household were listed and for each member having an episode of fever, further data was collected on contact with CHW, type of provider accessed for testing, confirmation of malaria through testing and type of provider accessed for treatment. In addition, CHWs of habitations covered under survey were contacted and the surveyors counted the quantity of RDTs and anti-malarial drugs (adult dosage of artemisinin-based combination and chloroquine) available with each CHW on the day of survey. 84 surveyors were trained for data collection.

Similar methodology was employed for all three rounds of survey, except that additional questions were asked in the 2018 round on the treatment-completion and whether it was under observation of concerned provider. Treatment completion was taken as 3 days of treatment with artemisinin-based combination or chloroquine. In order to confirm the reporting by malaria cases/families, the surveyors counted the empty ACT and chloroquine packs. Treatment under direct observation was taken as 3 days of treatment under direct supervision of provider. Data analysis involved descriptive comparisons for key indicators. Confidence intervals at 95% were reported for key indicators.

## Results

### Sample profile

Around half of the respondents were women. The average size of household was around 5. Around half of the individuals in studied households belonged to the Scheduled Tribes. Around 30% of the respondents had education of 8th standard or above (Table 2). The number of fever and malaria cases reported in the three rounds is shown in Table 3.

**Table 2:** Profile of sample households

Profile of household respondents	August, 2015	November, 2016	November, 2018
Average age of respondents (years)	41	40	40
Gender of respondent			
Male	1467 (44.8%)	1500 (49.2%)	1539 (36.2%)
Female	1808 (55.2%)	1544 (50.6%)	2718 (63.9%)
Not responded	2 (0.1%)	5 (0.2%)	0 (0%)
Household size (mean)	4.9	5.0	5.1
Caste (social group)			
Scheduled tribes	1907 (58.2%)	1419 (46.5%)	2408 (56.6%)
Scheduled castes	321 (9.8%)	303 (9.9%)	443 (10.4%)
Other backward classes (OBC)	802 (24.5%)	854 (28.0%)	984 (23.1%)
Others	194 (5.9%)	237 (7.8%)	189 (4.4%)
Not reported	53 (1.6%)	236 (7.7%)	233 (5.5%)
Educational status of respondents			
Class 8 or higher	826 (25.2%)	895 (29.4%)	1276 (30.0%)
Class 5–7	494 (15.1%)	439 (14.4%)	593 (13.9%)
Class 1–4	224 (6.8%)	184 (6.0%)	276 (6.5%)
No formal education but literate	381 (11.6%)	303 (9.9%)	474 (11.1%)
Not literate	1214 (37.1%)	1115 (36.6%)	1343 (31.6%)
Not reported	138 (4.2%)	113 (3.7%)	295 (6.9%)

**Table 3:** Number of fever and malaria cases

	August, 2015	November, 2016	November, 2018
Total number of fever cases	783	1151	1153
Total fever cases tested	671	1050	828
Total number of malaria cases confirmed out of those febrile individuals who got tested	216	379	250
No. of malaria cases received treatment	209	375	241

### Contact of fever cases with CHWs

In 2018, 62.4% of all fever cases contacted CHWs, which was greater than the proportion in 2015 (Table 4). The proportion of febrile children contacting CHWs was similar to that for other ages (Table 5).

**Table 4:** Proportion of fever cases who contacted mitanin-CHW, with CI

	August, 2015	November, 2016	November, 2018
No. of all fever cases	783	1151	1153
Proportion of all fever cases who contacted CHW (%)	56.4 (53.9–58.9)	68.5 (65.8–71.2)	62.4 (59.5–65.1)

**Table 5:** Proportion of under-5 child fever cases who contacted mitanin-CHW, with CI

	August, 2015	November, 2016	November, 2018
Number of under-five child fever cases	140	164	224
Proportion of under-five child fever cases who contacted CHWs (%)	63.1 (57.4–68.8)	67.1 (59.9–74.3)	64.0 (57.5–70.5)

### Coverage of fever cases in terms of RD testing by CHWs

Of the fever cases found in 2018, 72% had got tested for malaria. The share of different providers in testing of fever cases is given in Table 6. Out of the fever cases tested in 2018, 70.5% were tested by CHWs. This was nearly double of their share in 2015. In testing of fever cases, as the share of CHWs grew over the rounds, the share of private providers declined.

**Table 6:** Proportion of fever cases tested for malaria by type of provider, with CI

Indicators	August, 2015	November, 2016	November, 2018
Total number of fever cases who were tested	671	1050	828
Proportion (%) of different types of providers in fever cases tested			
CHWs	33.7 (31.5–36.0)	57.1 (54.2–60.0)	70.5 (67.1–73.9)
Government facilities other than CHWs	19.0 (17.0–21.0)	16.8 (14.6–19.0)	14.0 (11.7–16.3)
Private Providers	32.1 (29.8–34.4)	21.4 (19.0–23.8)	15.5 (12.9–18.1)

### Coverage in treatment of malaria by CHWs

Of the malaria cases found in 2018, 96% had received treatment. The share of different providers in treatment of malaria cases is given in Table 7. The share of CHWs in treatment of malaria cases in 2018 was 68.6% whereas it was 28.1% in 1st year of implementation i.e. 2015. In treatment of malaria cases, as the share of CHWs grew, the share of private providers declined sharply. CHWs and government facilities together contributed to around 82% of all malaria cases treated in 2018.

**Table 7:** Distribution of malaria cases according to the source of treatment, with CI

Treatment of malaria cases	August, 2015	November, 2016	November, 2018
Number of malaria cases treated	209	375	241
Source of treatment for malaria cases (%)			
Mitanin-CHWs	28.1 (23.8–32.4)	43.5 (38.5–48.5)	68.6 (62.7–74.5)
Government facilities other than CHWs	25.9 (21.7–30.1)	28.0 (23.5–32.5)	13.3 (9.0–17.6)
Private Providers	46.0 (41.2–50.8)	28.4 (23.9–32.9)	17.8 (13.0–22.6)

### Patient satisfaction with treatment

In 2018 survey, a question was asked from respondents regarding their satisfaction with treatment received (Table 8). Patient satisfaction was similar for all types of providers including CHWs.

**Table 8:** Proportion (%) of malaria cases reporting satisfaction with treatment in November, 2018 with CI

Proportion (%) of malaria cases satisfied with Treatment—by provider type	
CHW (n = 166)	93.9 (90.2–97.5)
Government facilities other than CHWs (n = 32)	96.9 (90.7–100)
Private (n = 43)	90.7 (81.9–99.5)

### Treatment completion

Treatment completion rate (at-least 3 continuous days of treatment) was greater for CHWs in comparison to other providers (Table 9). In terms of providing treatment under direct observation, CHWs were way ahead of other providers (Table 10).

**Table 9:** Treatment completion rate for malaria in November, 2018 with CI

Proportion (%) of malaria cases who received at-least 3 continuous days of treatment—by provider type	
CHW (n = 166)	89.1 (84.4–93.7)
Government facilities other than CHWs (n = 32)	62.5 (45.5–79.5)
Private (n = 43)	62.7 (48.1–77.3)

**Table 10:** Proportion of malaria cases who received treatment for at-least 3 days in November 2018, under Direct Observation of Provider with CI

Proportion (%) of malaria cases who received at-least 3 days of treatment under direct supervision of Provider—by type of provider	
CHW (n = 166)	72.3 (65.8–78.8)
Government facilities other than CHWs (n = 32)	9.7 (0–20.2)
Private (n = 43)	16.3 (3.4–29.2)

### Mortality among malaria cases

Number of deaths in malaria cases who were confirmed through testing is given in Table 11. The Malaria Mortality Rate in 2018 was 4.7 deaths per 100,000 population at risk. In 2018, there was one death amongst 250 malaria cases, i.e. a fatality rate of 4.0 per 1000 confirmed malaria cases.

**Table 11:** Deaths among malaria cases during 2015 and 2016 survey period

Indicators	August, 2015	November, 2016	November, 2018
Number of Malaria cases	376	385	250
Total Population	15,679	15,023	21,405
Number of malaria cases who died	2	4	1
Fatality rate of malaria cases (per 1000 cases)	5.3 (2.5–8.1)	10.4 (0.25–20.5)	4.0 (0–8.2)
Malaria Mortality Rate (Deaths per 100,000 population at risk)	12.8	26.6	4.7

### Availability of RDT and relevant anti-malarial drugs with CHWs

In 2015 survey, out of the 265 habitations covered, 259 (97.7%) had a CHW in place. The surveyors checked the availability of RDTs and drugs with the CHWs on day of survey. Table 12 shows that availability of RDTs and drugs improved over the three rounds.

**Table 12:** Proportion (%) of CHWs having RDT and anti-malarial drugs with CI

	August, 2015	November, 2016	November, 2018
No. of CHWs	259	250	348
Proportion of CHWs having RDT	81.8 (77.1–86.6)	95.0 (92.4–97.6)	96.1 (94.1–98.1)
Proportion of CHWs having Adult ACT	50.0 (43.9–56.1)	79.9 (77.5–82.3)	80.2 (78.1–82.3)
Proportion of CHWs having chloroquine	87.6 (84.7–90.5)	83.3 (80.8–85.8)	94.8 (92.8–96.8)

## Discussion

According to the quantitative assessment carried out in the current study, 62% of febrile cases in rural population of Chhattisgarh contacted CHWs in 2018. RDT, artemisinin-based combinations and chloroquine were available with 96%, 80% and 95% of CHWs, respectively. From 2015 to 2018, the share of CHWs in testing of febrile cases increased from 34 to 70%, while their share in treatment of malaria cases grew from 28 to 69%. CHWs performed better than other providers in 3-day treatment-completion and administered medication under direct observation to 72% of cases they treated.

A study in 2015 with mitanin-CHWs in Chhattisgarh had shown that CHWs can attain adequate skills [23]. Studies from many countries have shown that CHWs perform well in case management of malaria at community level. Close to 100% of CHWs studied in some interventions were found to be carrying out the correct diagnostics and treatment [12, 24, 25]. CHWs have been found to be good in adherence to RD tests [26]. However, most of these studies have made observations in interventions involving a small number of CHWs, newly trained for CCMM [12, 24, 25]. The current study did not include testing the skills of CHWs because earlier evaluations were available [8, 9, 23].

An important performance measure is the extent to which febrile cases in the population get covered. Household data collected in 2010 in two intervention districts of Cameroon where the CCMM programme was run for the preceding 1 year as a pilot project showed that for 51% of under-five children, CHWs were contacted when suffering from fever [18]. Another pilot study conducted in two of the malaria endemic districts of Kenya in 2009 after 1 year of intervention in 2008 reported that 34.6% of caregivers of under-five febrile children had approached CHWs, which was a significant increase from 2.1% in 2008 baseline study [19]. A study among mothers of under-five children in Ethiopia in 2003 found that 40% of the mothers who had fever approached a CHW first [20].

In the current study, the share of mitanin-CHWs in testing and treating malaria increased over the three rounds, while that of non-state providers shrunk. By the 4th year of intervention, the public-sector including the CHWs enjoyed more than 80% share in malaria testing as well as treatment. Studies have shown that healthcare for malaria causes significant out-of-pocket expenditure and financial risk for households [27]. Since the services of mitanin-CHWs were available at a close distance and free of charge for patients, there is a likelihood of reduction in out-of-pocket expenditure for malaria detection and treatment. Most of the individuals treated by CHWs, reported their satisfaction with the treatment.

The current study found that CHWs played an important role in ensuring completion of treatment for malaria. The method of measuring complete-treatment in this study satisfies the conditions of being called ‘definite adherence’ [28, 29]. This is another aspect where CHWs offered a big advantage over other providers. The CHWs were trained to ensure treatment under direct observation and it was facilitated by their proximity to patients. A systematic review has reported treatment-completion of 62–93% depending upon methods used [30]. A study from Sierra Leone reported 63% to 79% treatment-completion and it was 64% in a Tanzania-based study [29, 31]. The proximity of CHWs to patients and easy availability along with willingness to follow-up for treatment-completion can be important factors [32]. A study in Myanmar showed that CHWs were technically as good as other providers in managing malaria but offered the advantage of proximity [33]. In comparison to well-trained CHWs, a large proportion of local private-providers in LMIC contexts are untrained and though their services can also be convenient to access, the treatment given maybe inappropriate.

Another significant aspect was the equity in terms of coverage of vulnerable groups. The Scheduled Tribes are one of the most vulnerable social groups in India [34]. The 9 million population in which CCMM was implemented in Chhattisgarh consisted predominantly of the Scheduled Tribe communities.

Though the current study was not designed to assess the impact of CCMM on malaria-related mortality, it reports lower mortality for 2018 in CCMM covered population than some of the older estimates from other studies in malaria endemic regions of India [35]. What explains the increased coverage achieved through the CHWs in the current study? There could be many factors. The average population per CHW in the programme-area in Chhattisgarh was around 235 in year 2016 [6]. The average population per CHW in rural areas at all India level on the other hand was 919 [6]. Among all Indian states, Chhattisgarh state had the most favourable ratio of rural population per CHW [6]. The CHW programme in Chhattisgarh aimed to cover all rural habitations. The programme area had as many as 37,696 CHWs for 9947 villages. The design of CHW programme in Chhattisgarh was to select at least one CHW per habitation. The fact that a CHW was available in most of the habitations could also be an important factor in achieving increased coverage in malaria-management. The distance between the CHWs and the households was small. Each CHW was a resident of the habitation she covered. It made frequent contact possible without the need of transport arrangements. A study in Kenya had shown that CHWs looking after smaller populations performed better [19].

CHWs were able to attract the febrile cases because through them malaria-detection became accessible. It can be a key factor in tribal populations and remote geographies in which most of the malaria exists in India and other LMICs. Detection by CHWs has been a big part of the success achieved in malaria-management by another Indian state, Odisha [36, 37].

For CHWs to be successful in CCMM, uninterrupted and adequate supply of drugs and diagnostics for malaria are the essential inputs [19, 38, 39].Many studies from Africa have pointed out that while uptake of treatment increases with CHWs providing CCMM but inadequate supplies have often been the critical bottle-neck [40–42].

An Indian study showed that inadequate availability of rapid tests and drugs with CHWs can undermine community’s confidence in them [43]. An earlier study with mitanin-CHWs in Chhattisgarh state in India in 2015 had described the challenges faced by CHWs in surveillance of malaria that included the availability of diagnostics and drugs [44]. A study in Myanmar found that in the 3rd year after making ACT available in the country 77.7% of the anti-malaria working CHWs had availability of RDT, 83.1% had ACT while 67% had chloroquine [24]. What seems to have helped in the current study was the importance of ensuring supplies getting recognized and addressed in the preparatory phase leading to the programme.

A recent study using qualitative methods in Lao has reported the factors favoring community-participation in receiving mass drug administration for malaria—understanding of communities regarding rationale of the intervention, free services, cohesiveness in communities and their collaboration with CHWs and health authorities [45]. In the current study, the stakeholder-engagement and social-mobilization campaign in the preparatory phase could have facilitated the CCMM programme. Other factors favouring the CHWs in the current study could be that most of them were well-established in the communities for over a decade and the training effort was adequate and it built on their earlier knowledge and skills.

Acceptability of malaria interventions among the communities has also been highlighted as an important factor [40]. A systematic review found that the community-acceptability of malaria interventions through CHWs improved once CHWs started using RDTs. CHWs were able to give better care using RDT as it gave correct diagnosis immediately, allowing prompt treatment. RDT usage by the community further helped them save money on transport [40].

Social recognition, continuous training and frequent supervision, clear role definitions, financial and non-financial incentives have been found to be important for improving general performance of CHWs [46]. Studies specific to CHWs’ work on malaria have reported that apart from training and availability of essential supplies, length of experience since becoming CHWs, getting feedback and support from supervisors and communities were also important factors in their performance [29, 47, 48].

India has made significant progress in reducing malaria incidence in 2018 and 2019, led by two of the highest burden states—Odisha and Chhattisgarh [49]. Indian policy has begun to recognize the importance of achieving high coverage of early detection and full treatment and the role of CHWs in it [50]. Many regions in India and LMICs with high burden of malaria can benefit from a strategy based on well-equipped CHWs close to the community.

A study of country-wide CCMM programme in Burkina Faso reported findings for three consecutive years from 2011 to 2013. This household-reported data showed that 1% to 9% of the febrile under-five children were brought to the CHWs and that this scenario did not improve from 2011 to 2013 [21]. The study describes how large scale interventions are subject to the uncertainty of various social factors (e.g. relationship and communication between CHW and beneficiaries), the geographical factors (e.g. distance between the beneficiary and the CHWs or the nearest health care facility) and systemic factors (e.g. regularity of the drug supply to the CHWs) and may result in varying degree of outcomes different as compared to those of pilot studies [21]. The current study suggests that in large-scale programmes, systemic issues including the geographic availability of CHWs, training and support for CHWs, supplies of tests and drugs, incentives for CHWs and preparations made before launching the programme could play an important role in coverage apart from mechanisms of community-participation in health. Understanding the factors or processes contributing to success in scale-up of CCMM can be a useful direction for future research.

How ‘coverage’ is understood in context of malaria varies, depending upon the nature and scale of intervention. For example, coverage reported for Mass Drug Administration for malaria is different than coverage under Case-Management that involves testing before treatment [12, 22, 51, 52]. Small interventions conducted for the purpose of study often report better coverage within a smaller population than what large-scale programmes show. The smaller scale of studies limits their coverage in relation to overall population of provinces or countries [52]. This underscores the need for remaining conscious of such differences while comparing coverage achieved in different malaria programmes.

### Study limitations

The usual limitations of descriptive studies apply. There was no baseline available. The programme being a universal programme for all high-burden areas in Chhattisgarh, it was infeasible to get enough sample of population without CHWs to make a comparison. Given the quantitative nature of assessment in this study, it did not explore the reasons for increasing coverage. The study did not use inferential statistics or GIS based analysis, which could have helped in such exploration. The study did not capture data on social characteristics of communities covered by the programme or the CHWs. Future studies are recommended to address the above gaps.

There was a gap between the number of febrile persons contacting CHWs and other providers and those tested by them. One reason was that a proportion of providers did not have RDTs available with them. No data was collected regarding RDT availability with providers other than CHWs. The gap in testing by CHWs was greater in 2018 even though the proportion of CHWs having any RDTs was better. It could be the case that in 2018 most CHWs had some RDTs but not enough quantity to test every febrile person contacting them. The current study did not identify the reasons for the gap in testing.

The study did not include identification of complicated cases and referrals, though it was part of CCMM. The treatment definition did not include primaquine though the Chhattisgarh protocol required CHWs to refer the cases to formal healthcare providers for taking primaquine.

## Conclusions

CHW practice leading to desired results in CCMM has been recognized, though such studies have been limited to small programmes. This study shows that large-scale CCMM through well-trained CHWs, backed by supply of rapid-tests and drugs was able to achieve high coverage and treatment-completion rates. CHWs’ proximity to populations they cover offers a big advantage. This study adds to one of the crucial but relatively less reported area of CCMM implementation, i.e., the extent of coverage of the total febrile population by CHWs which subsequently determines the actual coverage of case management in malaria.

Further implementation-research is recommended to identify the underlying factors that can contribute to success of large-scale interventions in community based management of malaria.

## Data Availability

The datasets used and/or analysed during the current study are available from the corresponding author on reasonable request.

## Abbreviations

ACT: Artemisinin-based combination therapy
CCMM: Community Case Management of Malaria
CHW: Community health workers
LMIC: Low and medium income countries
RDT: Rapid diagnostic tests
SEAR: South-East Asia Region
